# The status of China’s international division of labour from Marx’s theory of international value

**DOI:** 10.1371/journal.pone.0312748

**Published:** 2025-01-24

**Authors:** Hui Ouyang, Huaibing Chen, Johnny Fat Iam Lam, Xianke Li

**Affiliations:** 1 Finance College, Jiangxi Normal University, Nanchang, R. P. China; 2 Business College, Jiangxi Normal University, Nanchang, R. P. China; 3 Faculty of Humanities and Social Sciences, Macao Polytechnic University, Macao, R. P. China; University of Maribor Faculty of Arts: Univerza V Mariboru Filozofska Fakulta, SLOVENIA

## Abstract

Based on Marx’s theory of international value, this paper analyzes the carrier of unequal exchange and the path of international transfer of surplus value, constructs an international division of labour status index by comprehensively observing the organic composition of capital in the export and import sectors, and tries to accurately grasp the characteristics of the current international division of labour pattern coexisting between horizontal products and vertical products. At the same time, based on the data of all available HS92 six-digit code categories in various countries in the world from 1993 to 2016, the level of international division of labour in China is empirically analysed. The results show that: (1) there is no breakthrough in the situation of China’s low status of international division of labour; (2) the high capital-organic composition of the import sector is the main reason for the low status of international division of labour; (3) the continuous decline in the absolute value of captured surplus is a global phenomenon; (4) WTO accession has not significantly improved the status of international division of labour. Finally, the idea of improving the status of international division of labour through the development of domestic regional cooperation is put forward.

## 1. Introduction

Accurately measuring a country’s position in the international division of labour system is crucial for the formulation of evidence-based foreign trade policies. However, keeping up with the dynamic trends in the international division of labour poses a challenge to statistical activities, as existing databases are inadequate to reflect each country’s status in this regard. With the advancement of information and communication technologies, the deepening of trade liberalisation and political developments [1], production tasks and activities have become increasingly "internationally dispersed", leading to the emergence of "borderless production systems". Trade in goods or services has been transformed into trade in tasks, where the organisation of production extends beyond national borders through "global production networks" [2,3] and the creation of value extends beyond national borders through "global value chains" [4]. The international division of labour is evolving from horizontal product-based divisions to vertical product-based internal divisions. More and more products are crossing national borders to be integrated into borderless production systems organised by global production networks, while value is added within global value chains. However, customs statistics on trade flows still rely on traditional product-based approaches across different countries without identifying the size of their respective value chains. This leads to problems such as "double counting" and "statistical illusion" [5,6], which inevitably distort the real dynamics of the division of labour and trade interests between countries [7,8]. This has significant implications for a nation’s long-term development strategy.

To address these challenges, we propose the construction of an international division of labour index that accurately assesses a country’s position within the network era of international division of labour, characterised by both horizontal product specialisation and vertical product integration. In Marxist theory, the value of a commodity is determined by the amount of socially necessary labour time required for its production. This implies that the value of a commodity is created by labour, and that the value of a commodity is equal to the amount of socially necessary labour time required for its production. The remuneration paid to workers by capitalists is solely in accordance with the value of their labour, which is to say, wages. Only a portion of the total labour time of the worker is dedicated to the production of value, with the remainder employed in the generation of surplus value for the capitalist without compensation. This is the source of surplus value within a country. The advent of international trade and globalisation has resulted in workers being required to produce surplus value not only for capitalists in their own country, but also for capitalists in other countries. This is known as the international transfer of surplus value. The international value of the exchange of commodities is contingent upon the labour time required by the international community when commodities from disparate countries are introduced into the international market. The same commodity is subject to two distinct standards of national and international value. Conversely, countries with higher productivity, whose national labour is regarded as more intensive labour in the global market, can obtain a higher international value, thereby facilitating the transfer of surplus value from one country to another. The capitalist international division of labour has facilitated the exponential growth of capitalist productive forces, reinforced the specialisation of countries, elevated global productive forces, and disseminated the advanced capitalist mode of production and modern civilisation. However, the international division of labour of capitalism reflects the capitalist relations of production, and the transfer of international surplus value also gives rise to inequality in the international division of labour. This paper is based on Marx’s theory of international value and seeks to analyse and examine the inequality of the international division of labour.

This paper makes three potential contributions. First, it develops an index of international division of labour status by considering the organic capital composition in export and import sectors. Second, drawing on Marx’s theory of international value, this study empirically examines changes in China’s international division of labour status from 1993 to 2016 and confirms a solidifying trend in the integration of developing countries into the global value chain system. Finally, it also shows that embedding in the global value chain system facilitates the economic development of developing countries as their captured surplus diminishes with increasing economic globalisation. The structure of the paper is as follows. In Section 2, we first review the relevant literature. In Section 3, the measurement principle of international division of labour based on Marx’s theory of international value is discussed in detail and the index of international division of labour is constructed accordingly. In Section 4, the empirical analysis of the index is carried out to measure China’s international division of labour status. Finally, in section 5, we summarise the research of this paper and put forward specific countermeasures and suggestions.

## 2. Literature review

In early research on international division of labour, the measurement method relied primarily on traditional levels of division of labour between products. Scholars occasionally used the observation of indices such as revealed comparative advantage (RCA), revealed competitive advantage (CA) and trade competitiveness (TC) to conduct research and make judgments on specific product types. The underlying principle is that these products typically represent the comparative or competitive advantages of countries with a high degree of international division of labour; thus, the greater the number of advantages in a product, the higher the degree of international division of labour [9]. However, this method focuses only on individual products or general product categories. As a result, scholars have attempted to consider all export products comprehensively [10,11], but they still fail to account for intermediate products, leading to problems such as double counting and incomplete observations.

An increasing number of scholars have come to realise that due to the changing dynamics of the international division of labour, traditional analytical tools such as the competitiveness index based on specific products or industries no longer accurately capture the true nature of the international division of labour. As a result, scholars have begun to explore methods of accounting for value-added trade. The index of vertical specialisation rate was first proposed by Hummels et al. (1999) to measure the real level of foreign trade between countries [12], in order to overcome the drawback of not taking into account the value of intermediate products. On this basis, Johnson and Noguera (2012) modified several assumptions and proposed an analytical framework for the value-added export ratio [13]. Later, Koopman et al. (2010) established a relatively mature method of measuring the overall participation and division of labour status index of the global value chain after decomposing the export into five items [8], which has led to numerous empirical studies based on this framework [14–16].

The Koopman (2010) method (KKW) is based on the principle of comparing upstream participation indicators with downstream participation indicators [8]. The upstream participation indicator refers to the share of domestic intermediate products in a country’s total exports, while the downstream participation indicator refers to the share of intermediate products from other countries in a country’s exports. If a country’s upstream participation index is higher than its downstream participation index, it indicates that the country operates in the upstream value chain and has a higher level of international division of labour. However, a drawback of the KKW method is that it makes a subjective judgement as to whether value is added upstream or downstream, assuming that being upstream necessarily implies a high level of international division of labour. This overlooks situations where being either upstream or downstream may simply reflect the structure of the product process, with no inherent correlation to the level of technological sophistication. For example, a country that is only a supplier of raw materials may be classified as upstream but not necessarily as having a high international division of labour. It should also be noted that the KKW method does not cover final products, but only intermediate products, which makes it incomplete.

To address the limitations of the KKW method, Liu (2017) made improvements by introducing two indicators: the proportion of domestic value added in total exports and the proportion of other countries’ value added in total imports [17]. A higher proportion of domestic value added indicates a higher level of division of labour. Furthermore, Xing & Li (2018) proposed a bilateral nested status index as an alternative approach to improve the KKW method [18]. This enhanced approach circumvents the constraints inherent to the KKW methodology. The method not only eliminates the bias present in the upstream and downstream sectors, but also incorporates the final consumer goods, thus providing an appropriate means of measuring the division of labour within the global value chain. However, it should be noted that not all products are included in the global value chain. Irrespective of the advancement of productive forces, there will always be products whose intrinsic characteristics render them unsuitable for the requirements of the production process segmentation.

The current pattern of international division of labour has a network structure that includes both horizontal and vertical division of labour. While the latter cannot replace the former, they must co-exist. However, these two methods have their limitations. Furthermore, they effectively address the issue of subjective judgement in upstream and downstream relationships; however, if several economies have similar technological capabilities, different outcomes may result depending on each country’s position in the supply chain, thereby increasing risk.

Scholars have also proposed alternative approaches to improvement. First, they suggest looking at export prices, arguing that a country’s position in the international division of labour can be inferred from the gap between its export prices and the global average price. The larger the gap, the higher the country’s position in the international division of labour [19]. However, using export value alone to measure the status of the international division of labour is obviously unconvincing, as it fails to capture both the dynamics of the revenue-cost structure and the characteristics specific to different time periods. Second, scholars propose to assess the level of productivity, claiming that higher productivity corresponds to a more advanced international division of labour status [20]. While using overall productivity levels as an indicator is acceptable, exporting products without comprehensive database support is not. Third, an input-output analysis is suggested to determine how exports of high-tech products drive domestic value added. A stronger pull effect indicates a higher international division of labour status [21]. However, it remains debatable whether this pull effect really reflects the state of the international division of labour; rather, it primarily shows the dependence of domestic production on high-tech exports. As long as structural differences between domestic and foreign demand are not taken into account, it cannot serve as an accurate indicator of the state of the international division of labour.

From the literature reviewed above, scholars have extensively discussed the construction of measurement methods for assessing the status of the international division of labour; however, these discussions are not without their limitations. Building on existing research and drawing on Marx’s theoretical method, this paper aims to accurately capture the characteristics of the network era of the international division of labour, which is characterised by simultaneous horizontal product specialisation and vertical product fragmentation. Using comprehensive commodity data classified by HS92 six-digit codes from 1993 to 2016, this paper conducts a comparative analysis to examine China’s international division of labour in both vertical and horizontal dimensions. To the best of our knowledge, this study is pioneering in considering the accumulation and distribution curves of the organic composition of capital within the export and import sectors while constructing them to assess the status of the international division of labour.

## 3. Framework of analysis

### 3.1. Overview of Marx’s theory of international value

The international division of labour status is the product of unequal exchange. If all international trade were based on equal exchange, there would be no concept of international division of labour status. Because the division of labour status of all countries would be at the same level. It is the unequal exchange hidden behind the appearance of free trade that determines how much a country gains in the international division of labour system. International trade may have twists and turns in practice, but, developed countries continue to promote free trade as the dominant paradigm and actively construct an international system of labour division. This is due to the fact that, within the context of globalisation, it is able to engage in unequal exchange on the basis of its substantial capital and sophisticated technology. Conversely, it can maintain its elevated status within the global division of labour by exerting control over the regulations governing international trade and international finance, thereby securing a greater share of international surplus value transfer. Amin (1977) posits that developed countries incorporate other countries into their structure through capital accumulation and external expansion, thereby forming an unequal dependency relationship [22]. The phenomenon of globalisation can be defined as the globalisation of capitalism [23]. Developed capitalist countries not only export goods, services, financial assets and technology to the global market, but also export the specific relations of production that underpin these activities. It is erroneous to view the development of capitalism as a movement within an isolated and closed system [24]. Furthermore, while the movement of goods and capital across national borders is relatively unrestricted, the movement of labour is often subject to various types of restrictions and obstacles. The process of globalised production entails a shift in production activities from high-wage countries to low-wage countries, which presents the phenomenon of global labour arbitrage. Multinational enterprises reduce costs and enhance profitability by replacing domestic high-wage labour with foreign labour that is less expensive. This results in an imbalance between the mobility of production and labour, which in turn leads to further distortions in global wage differentials and exploitation rates [25]. Developed countries have demonstrated a robust commitment to safeguarding this additional benefit derived from unequal exchange. Such actions were undertaken in ways that were arguably more extreme and overt during the colonial era. However, with the advent of globalisation and the ascendancy of neoliberal cultural hegemony, this process has become increasingly peaceful and opaque.

Over the past three decades, the overwhelming majority of governments around the world have pursued extensive neoliberal economic policies [23]. The rationale is that although developing countries are only able to engage in the international division of labour in a passive capacity, they are nevertheless able to realise the value of domestic production factors, including labour, capital and land. This enables them to obtain some form of primitive accumulation, despite the difficulties associated with organising domestic social large-scale production. International trade confers tangible benefits on both developed and developing countries. However, for developing countries that are merely passive participants in the international division of labour system, there is an opportunity cost associated with such participation. Indeed, participation in the international division of labour system effectively entails the abandonment of the domestic division of labour system. The international division of labour system is dominated by developed countries, and the mechanisms that promote technological progress, such as specialisation, competition and knowledge spillover, are ineffective within this system. It is challenging for developing countries to undergo evolutionary and upgrading processes through this system. In other words, developing countries are susceptible to becoming enmeshed in an inferior international division of labour. Concurrently, the intrinsic merits of the domestic division of labour system remain unestablished, necessitating continued reliance on the international division of labour system. This illustrates an imbalance between the domestic and international division of labour systems. It is challenging for developing countries to advance in this context, which is illustrative of the middle-income trap. It can be seen, therefore, that the middle-income trap has its roots in the trap of the international division of labour, which in turn has its roots in the low status of the international division of labour, which in turn has its roots in the passive participation of developing countries in the international division of labour system. The latter is a consequence of unequal exchanges.

The essence of the status of international division of labour is a kind of international production relations characterized by unequal exchange, which is naturally determined by the level of productive forces. The term ’productive forces’ is used to describe the capacity of humans to subdue, alter and utilise the natural world for their own benefit, reflecting the complex and nuanced relationship between humanity and the natural environment. However, from the perspective of production results, it can typically be represented by the number of products produced per unit time or the amount of services provided. This is known as productivity. Furthermore, from the perspective of the production process, the productive forces can also be reflected in the tools or technologies employed in the production of material goods. To illustrate, the ratio of means of production to the labour force represents the technical composition of capital. In general, the composition of capital technology determines the proportion of constant and variable capital, that is to say, the composition of capital value. Marx designated this value composition of capital, which is contingent upon the technical composition of capital and can reflect the alterations in the technical composition of capital, as the organic composition of capital [26]. The organic composition of capital is a term peculiar to Marxian economics. Mainstream economics uses things like capital intensity. In terms of performance, the organic composition of capital is not very different from capital intensity. But the organic composition of capital treats both means of production and labour as capital. One is constant capital and the other is variable capital. By comparing the two, the source of surplus value and the degree of exploitation can be clearly observed. In contrast, concepts such as capital intensity in mainstream economics do not consider labour as capital. However, both reflect to some extent the level of technology in the production process. Globally, the heterogeneity of the level of productive forces across countries is evident. This is reflected in the gap between developed and developing countries. The quantity of a product or service produced per unit of time differs between the two, as do the value shares of the means of production and labour used in production. These reflect the great difference in the relationship between man and nature in the conquest and transformation of nature. This has laid the material foundation for unequal relations of exchange between people.

The occurrence of unequal exchange is based on the international transfer of surplus value. Marx believed that one country can continuously seize a part of the surplus labour of another country without paying any price for it [27]. This is because the formation mechanism of international value and national value is not the same. According to Marx’s labour theory of value, value has social attributes and reflects the relationship between people. The formation of value requires the connection between members of society and the recognition of the standard of socially necessary labour time. It is a process of transformation from concrete labour to abstract labour and from private labour to social labour [26]. These two processes of transformation require the free circulation of the factors of production. Otherwise there would be no connection between members of society and no certainty about the standard of socially necessary working time. Within a country, both transformation processes are relatively perfect, resulting from the relatively free circulation of production factors within a country. On a global scale, however, neither transformation is complete. The reason is that the originally unified international market is divided by individual nation-states, and the flow of production factors such as natural resources, labour, capital and technology between countries is not free, which inevitably hinders the socialisation on which the formation of international values depends. After the formation of national value, the game is played according to the organic composition of each country’s capital to form international value.

Therefore, Marx believed that the necessary labour time of the international society could not simply be deduced from the necessary labour time of the domestic society, but could only be measured by the average of the average labour intensity of each country. The average labour intensity varies from country to country; some countries are higher, others lower. The average of the countries then forms a ladder, and its unit of measurement is the average unit of world labour. Therefore, the more intensive national labour will produce more value at the same time than the less intensive national labour, and this will show up in more money. The more developed capitalist production is in a country, the more the intensity and productivity of national labour there exceeds the international level. Therefore, different quantities of the same commodity produced in different countries during the same labour time have different international values and therefore appear as different prices, i.e. as different amounts of money according to their respective international values [22]. The reason why the intensity and productivity of national labour increases with the advance of capitalist production is that the more productive national labour is also counted as more intensive labour on the world market, provided that the more productive countries are not forced by competition to reduce the selling price of their commodities to a level corresponding to the value of the commodities. [28] In other words, the national value is established first, and then the game is played according to the productivity of labour or the organic composition of capital in each country. Only in this way can the international value be formed.

This two-step model is very different from the one-step model in which the factors of production can circulate freely internationally. Through the natural borders of the nation-state, high-tech means of production are firmly monopolised in the hands of a few developed countries. On the surface, the establishment of the international division of labour system shows the transnational spread of socialised mass production, but it only spreads the fundamental capitalist contradiction between socialised mass production and private ownership of the means of production to the whole world, and also spreads the predatory behaviour of surplus value from one country to the whole world. The national value and the international value of the products exported by the developed countries are often very different. The domestic value is low, but the international value is high. Developing countries export products with a small difference between the national value and the international value. The reason is that the organic composition of capital in developed countries is high, while that in developing countries is low, and the two have different forces in the game of international value formation. Therefore, developed countries make use of the special mechanism of international value formation and the advantage of high organic composition of capital to export products with low domestic value but high international value, import products with high domestic value but low international value, and realise the international transfer and unequal exchange of surplus value.

Although factors such as technological monopoly power, military power, financial hegemony and labour markets can also cause unequal exchange, they are rather the result of unequal exchange, or at least the synergistic effect of unequal exchange. In particular, Emmanuel (1974) believed that in addition to the organic composition of capital, the unequal exchange caused by different wages was more worthy of emphasis [29]. However, the essence of wages is the value or price of labour. Marx believed that wages are not what they appear to be on the surface, not the value or price of labour, but only the disguised form of the value or price of labour [26]. Wages are the product of labour becoming a special commodity under the capitalist system of employment, and they are the embodiment of capitalist relations of production. Therefore, wages are not an exogenous variable, but are determined by the capitalist mode of production and ultimately by the organic composition of the productive forces or capital. In other words, it is not the difference in wages between countries that leads to unequal exchange, but the unequal participation in exchange that determines the different wage levels between countries. In addition, Ricci (2021) also considered the impact of profit margin differences between industries when analysing industrial value transformation by constructing Marx’s exchange rate determination model [23]. However, according to the law that an equal amount of capital produces an equal amount of profit, the difference in profit margin between industries is precisely caused by the different organic composition of capital in each industry. Therefore, if the organic composition of capital and wages and the rate of profit are included in the analytical framework in the examination of unequal exchange, it is impossible to remove the complex relationship between the organic composition of capital and wages and the rate of profit. This will to some extent affect the analysis.

From the above analysis, we can see that due to the special mechanism of international value formation, the organic composition of capital has become the basis for judging the international transfer direction of surplus value. In other words, surplus value is transferred from countries with low organic composition of capital to countries with high organic composition of capital. The international transfer of surplus value is the way to realise the unequal exchange, and the unequal exchange is the embodiment of the status of the international division of labour. Therefore, by observing the level of organic composition of capital in each country, it should be possible to judge the status of each country in the international division of labour system, and it is also in line with the law that productive forces determines the relations of production. This is based on Marx’s theory of international value to measure the status of the principles and ideas of the international division of labour.

### 3.2. Construction of division status index (DSI)

The observation of the organic composition of national capital should be based on the import and export sectors. Since international trade does not cover all commodities in the world, only those commodities that are imported and exported are included in the international division of labour system, and those commodities that are only produced, circulated and consumed in the country need not be considered. It is therefore necessary to examine the organic capital composition of the industries involved in the import and export of commodities in different countries. Furthermore, one country’s exports must be another country’s imports; they are different sides of the same thing. Globally, exports must equal imports, so it is possible to examine only the export sector.

To measure the capital components of each country’s export sector, we can first measure the capital components of the industries involved in a single commodity, and then integrate all export commodities. The organic composition of capital refers to the value composition of capital, which is determined by the technical composition of capital and can reflect the change in the technical composition of capital. It is expressed as the ratio of the value of constant capital to the value of variable capital, i.e. the ratio of the value of the means of production to the value of labour. However, due to the unavailability of data on constant and variable capital, it is difficult to quantify it directly on the basis of the original concept of the organic composition of capital.

In measuring the capital organic composition of the industry involved in a single commodity, this paper refers to the method of Fan Gang et al. (2006) to show the value added of technology [10] and the method of Hausmann et al. (2007) to improve the measurement [11]. Both may use different indicators, but the principle is the same and both are used to quantify the level of technology in a given good. They all indirectly reflect the technical relationship in which the worker controls the means of labour. To a certain extent, they reflect the ratio between the quantity of means of production and the quantity of labour using these means of production, which is closer to the concept of the organic composition of capital.

The specific rationale is as follows. Firstly, goods for which more technology is used in the production process. In the so-called technology-intensive goods, more means of production are invested and the organic composition of capital in the industry is higher. Secondly, according to Marx’s theory of international value, if a country wants to obtain more international surplus value transfer, it must use industries with a high organic composition of capital in its own country as much as possible, and choose to produce those commodities whose national value is much lower than the international value for export. The higher the organic composition of capital in the production process of a commodity, the lower its national value and the more likely it is to achieve international surplus value transfer. A country will then give priority to the production and export of those goods in which it has the greatest international technological advantage. Countries with more technological factors are more likely to produce and export more technology-intensive goods. Thirdly, countries with a high level of technological advancement have historically occupied a leading position in the international division of labour, specialising in the production of technology-intensive goods. Those countries with inadequate technical capabilities are constrained to select from the remaining options for the division of labour. The findings indicate that countries with a wealth of technological resources tend to possess a comparative advantage in skill-intensive goods, whereas countries with limited technological capabilities tend to excel in the production of other goods. Both groups of countries demonstrate a capacity for comparative advantage. The distinction is merely one of active and passive roles.

In accordance with the aforementioned principle, From the perspective of the global economy, the export of a particular commodity by a country with a comparative advantage in that commodity is more likely to occur when that country has a greater abundance of technological factors. This is because countries that export a given commodity are more likely to be dominated by countries with abundant technological factors. Consequently, the production process of the exported commodity will involve a greater intensity of technology and a greater input of means of production. The higher the organic composition of capital. Accordingly, a higher index of the organic composition of capital can be attributed to the commodity. This represents the fundamental valuation principle of the organic composition of revealed capital, which is characterised by two distinct attributes. One such instance is the absence of a necessity to directly estimate the organic composition of capital based on the proportion of non-capitalised and variable capital. It is sufficient to estimate this based on the comparative advantage of the commodity in international trade and the overall technological factor abundance of the country that shows comparative advantage in the commodity. Secondly, the revealed capital organic composition does not estimate the actual capital organic composition of the industry engaged in the production of a given commodity. The index is employed to facilitate a comparison of the organic capital composition of the industries engaged in the production of a given commodity. It can therefore be concluded that the organic capital composition of one industry in relation to another is either higher or lower.

The formula is as follows:

$$CCI_{j}=\sum_{i=1}^{n}\omega_{ij}\ln(A_{i})$$ (1)

CCI_j_ is the Capital Components Index of the industry involved in commodity j. A_i_ represents the index of technological factor abundance of country i. This is a measure of technology. The concept of total factor productivity can be employed to represent the technological factor abundance of a country. An increase in total factor productivity is indicative of a greater abundance of technical factors. It is challenging to calculate the total factor productivity of all countries based on the current database type and its availability. One potential solution is to approximate the replacement of total factor productivity with labour productivity. Labour productivity is typically expressed as the value added per worker. GDP per capita can be employed as an indicator for comparing labour productivity across countries; however, this approach ignores the differences in population structure. n is the number of countries in the world; ω_ij_ is the weight of country i’s comparative advantage in the export of product j, where:

$$\omega_{ij}=\frac{RCA_{ij}}{\sum_{i-1}^{n}RCA_{ij}}$$ (2)

RCA_ij_ is the comparative advantage of country i in commodity j, obviously with $\sum_{i=1}^{n}\omega_{ij}=1$, and with:

$$RCA_{ij}=\frac{x_{ij}/\sum_{i=1}^{n}x_{ij}}{\sum_{j=1}^{m}x_{ij}/\sum_{j=1}^{m}\sum_{i-1}^{n}x_{ij}}$$ (3)

Where x_ij_ is the export value of country i in commodity j; m is the number of goods. It can be observed that the organic composition index of industrial capital is represented by a weighted average of labour productivity. The weight represents the proportion of exports from each country in the industry as a whole in the global market. The weighted average of labour productivity allows the concept of socialised mass production and its associated production relations, as set out in the establishment of an international system of labour division and the formation of international value, to be reflected.

We acknowledge that a country’s exports encompass more than just commodity j. However, employing any form of weighted average method in the comprehensive approach may not be suitable due to the absence of a clear substitution relationship between the capital organic composition index of commodities. It is impractical to equate the capital organic composition index of commodity A with that of commodity B using a fixed monetary value. The worthiness of an infinite number of commodities A cannot be simply determined by comparing them to the organic composition index of capital for a single commodity B. In constructing the technical height index, we can refer to Fan’s (2006) comprehensive method [10], which is briefly outlined as follows.

**Fig 1:**
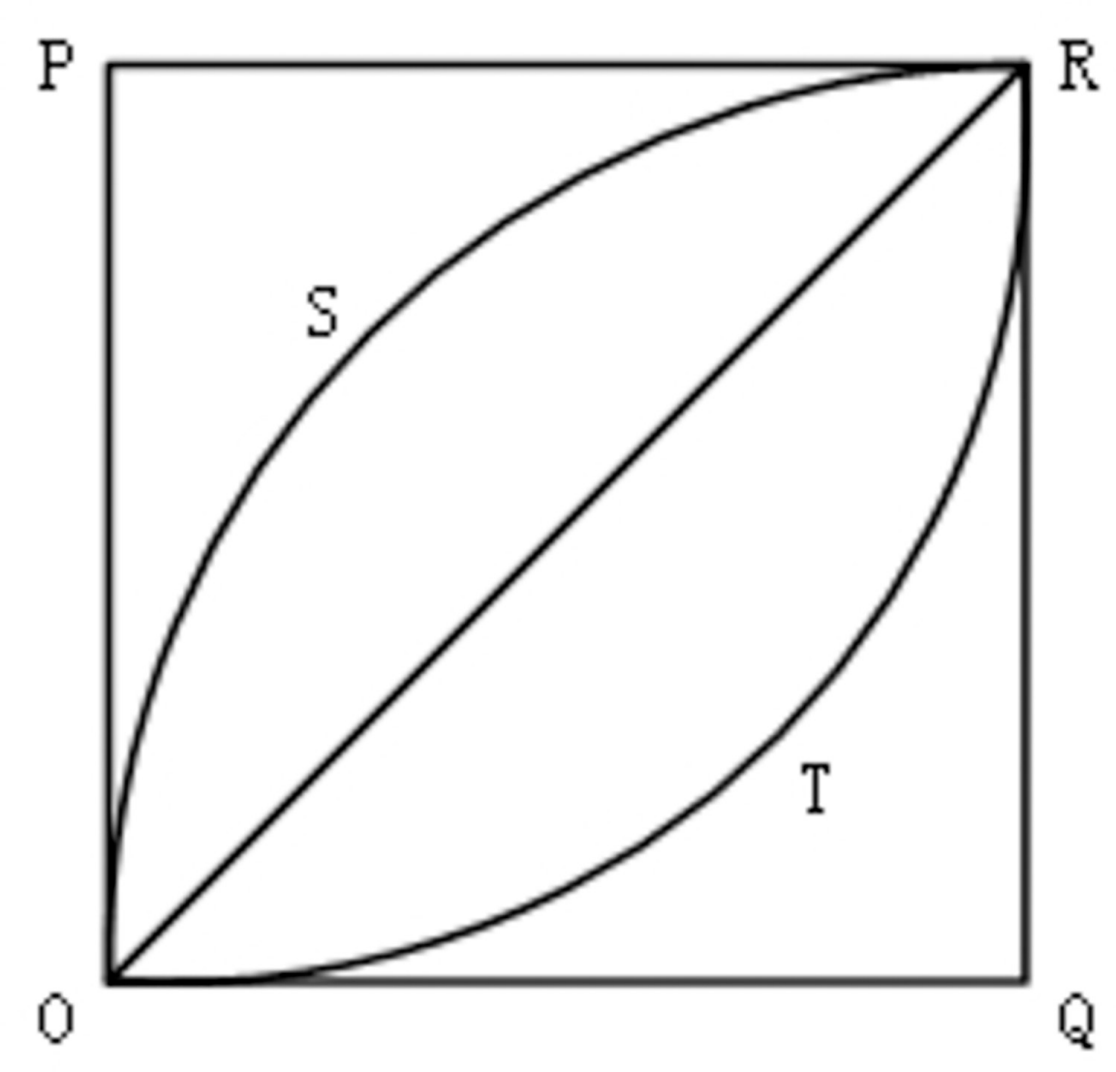
The cumulative distribution curve of the organic composition of capital.

Draw a coordinate axis, the horizontal coordinate represents m kinds of commodities, according to the capital organic composition index from low to high, its value is scaled equally, so that between 0 and 1; The ordinate represents the cumulative share of the export, which is also between 0 and 1. A curve similar to Lorenz curve can be obtained by drawing a line graph based on the data. According to the shape of the curve, the size of the capital organic composition index of the export sector can be judged. As shown in Fig 1, if the curve of country A is OPR, it means that only the commodities with the lowest organic composition index of capital are exported. If the curve of country E is OQR, it means that only the commodities with the highest organic composition index of capital are exported. If the curve of country B is OSR, the slope of the OS segment is greater than that of the SR segment, indicating that the cumulative share of low-capital organic composition commodities grows faster, indicating that the country mainly exports low-capital organic composition commodities. If the curve of country D is OTR, the slope of TR segment is greater than the slope of OT segment, indicating that the cumulative share of high capital organic composition commodities grows faster, it can be seen that the country mainly exports high capital organic composition commodities. If the curve of country C is OR, the slope is the same everywhere, and the cumulative share of the commodity with either low or high capital organic composition increases as fast, indicating that the export of goods is completely balanced, and the proportion of all types is the same. From the above analysis, we can see that the organic composition index of capital in the export sector of country E is the highest, followed by country D, country C, country B, and country A is the lowest. Therefore, it can be concluded that the closer the curve is to OQR and the smaller the covered area is, the higher the organic capital composition index of the export sector is. The formula for calculating this area is:

CCI_exi=1−∑t=1m∑j=1t[1−(xij/∑j=1mxij)]m (4)

The capital organic composition index (CCI_ex_i_) of the export sector in country i ranges from 0 to 1.

In the traditional international trade characterized by a horizontal division of labour among different products, it was feasible for CCI_ex_i_ to represent the position of Country i in the global labour division system. At that time, all the goods that countries exported came from within. In other words, the constant capital and variable capital used by countries to export goods are also from the domestic. Therefore, the organic composition of capital in the export sector can fully reflect the real situation of the country. However, with the development of productive forces and the advancement of globalization, the international division of labour has undergone major changes: from the original horizontal division of labour among products to the evolution of vertical division of labour within products, forming the global value chain. At this time, if the organic composition index of capital in the export sector is used to measure the status of international division of labour, there will inevitably be a statistical fallacy. For example, the developing countries mainly processing trade, their exports contain the technological added value of developed countries, so the organic capital composition index of the commodity is inevitably very high. However, the reality is that the division of labour in this country is not high.

In order to construct an accurate international division of labour status index, it is necessary to grasp and reflect the new situation of the international division of labour system, and it is necessary to combine the horizontal division of labour between products and the vertical division of labour within products. According to Marx’s theory of international value, if developed countries want to transfer international surplus value from developing countries, they must rely on such a method: exporting commodities with high organic composition of capital and importing commodities with low organic composition of capital. The exchange of high capital organic composition commodities for low capital organic composition commodities is not to produce low capital organic composition commodities, but to obtain international surplus value. International surplus value cannot be obtained by exports alone; it must be imported. In addition, looking at the organic capital composition index of the export sector alone, it is impossible to judge the amount of international surplus value it has obtained, and it is necessary to compare the export sector with the import sector to see which sector has the higher organic capital composition index. If the export sector is high, it means that international surplus value can be obtained. The higher the degree, the greater the amount of international surplus value obtained. Similarly, for developing countries, the extent to which their surplus value is captured cannot be judged by looking at the export sector alone. For example, in the context of vertical division of labour within products, developing countries dominated by processing trade export a large number of goods with high capital organic composition, but at the same time import a large number of goods with even higher capital organic composition. Such a comparison, it can be seen that its international division of labour status is not high. In short, in the context of the international division of labour system in which the horizontal division of labour among products and the vertical division of labour within products coexist, the flow direction and size of the international surplus value transfer should be examined at the same time, the organic capital composition level of the export sector and the import sector.

Based on this, we can utilize the individual capital organic composition index and import/export volume data of various commodities to draw the ’Lorenz curve’ for both the export and import sectors of a country. By analyzing and comparing the differences between these curves, as well as examining the area enclosed by the horizontal coordinate, we can obtain valuable insights into its international division of labour status.

**Fig 2:**
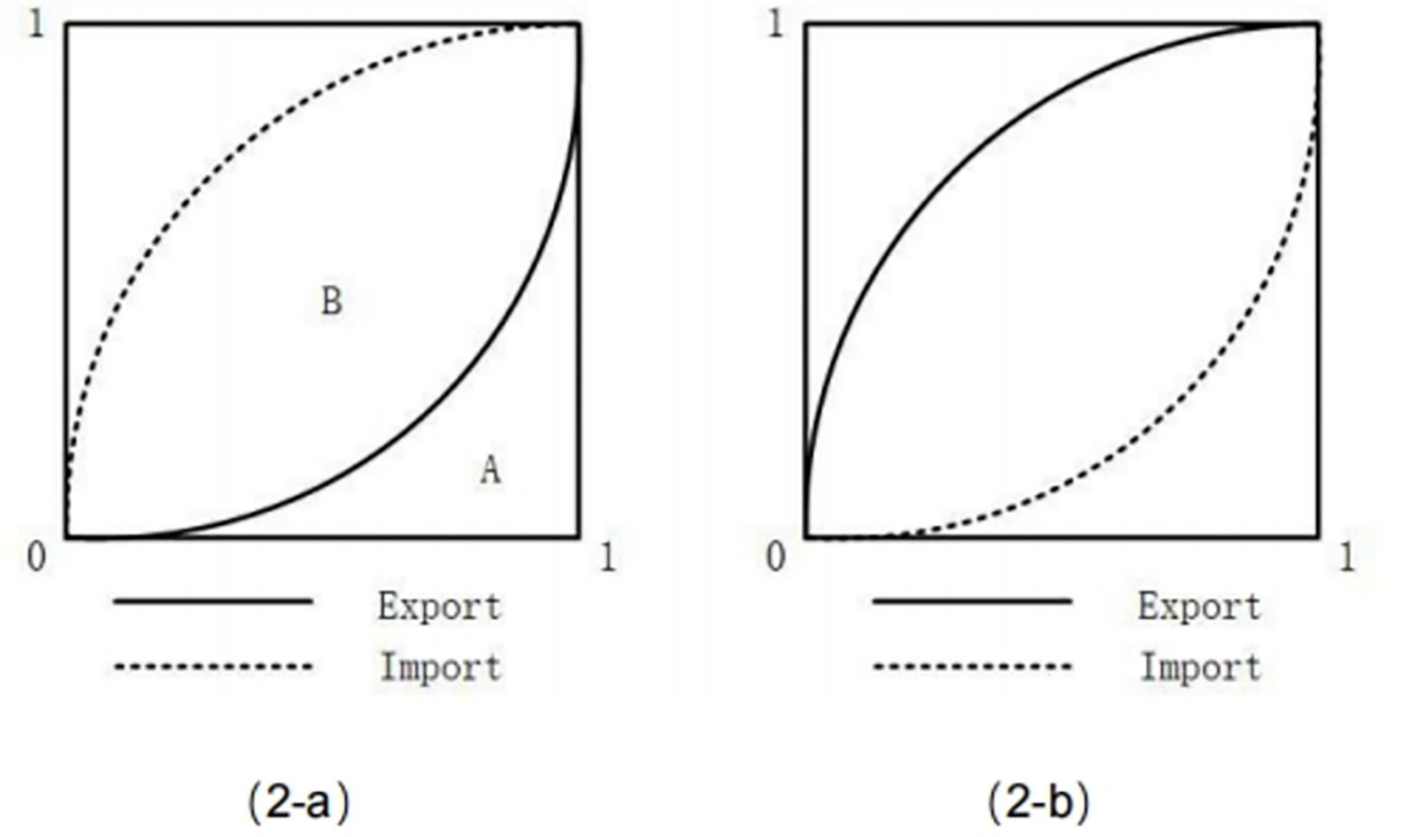
Types of labour division status.

The Fig 2 illustrates two distinct labour division statuses. In (2-a), the organic capital composition index of the export sector surpasses that of the import sector, indicating a high international division of labour status for the country as it can transfer surplus value from other nations. Conversely, in (2-b), the organic capital composition index of the export sector is lower than that of the import sector, suggesting that surplus value is transferred from this country by others and its international division of labour status is low. The ability to transfer international surplus value can be quantified by calculating the ratio between area B and area A. Here, area A represents CCI_ex_i_, which denotes the index of organic capital composition in the export sector; while area B signifies CCI_im_i_, representing the difference between organic capital composition indexes in both sectors.

CCI_imi=1−∑t=1m∑j=1t[1−(uij/∑j=1muij)]m (5)

Where u_ij_ is the import amount of countryi in commodity j. And the ratio of area B to area A is called the international division of labour status index (DSI), and its value is:

DSIi=∑t=1m∑j=1t[1−(xij/∑j=1mxij)]−∑t=1m∑j=1t[1−(uij/∑j=1muij)]m1−∑t=1m∑j=1t[1−(xij/∑j=1mxij)]m (6)

The range of DSI_i_ is [−1, ∞). Generally, a higher value signifies a more pronounced international division of labour status. A positive value implies the potential for acquiring international surplus value, while a negative value suggests that surplus value is captured by other countries. As evident from Formula (6), the index for international division of labour status is directly proportional to the disparity in organic composition index between import and export sectors’ capital and inversely proportional to the capital organization index in the export sector. The accumulation distribution curve graphically illustrates that area B represents the magnitude of capturing international surplus value by Country A through differences in organic composition index between import and export sectors, whereas area A denotes the cost incurred by Country A when capturing such surplus value. The ratio of area B to area A can be considered as an indicator reflecting Country A’s efficiency in capturing international surplus value.

**Fig 3:**
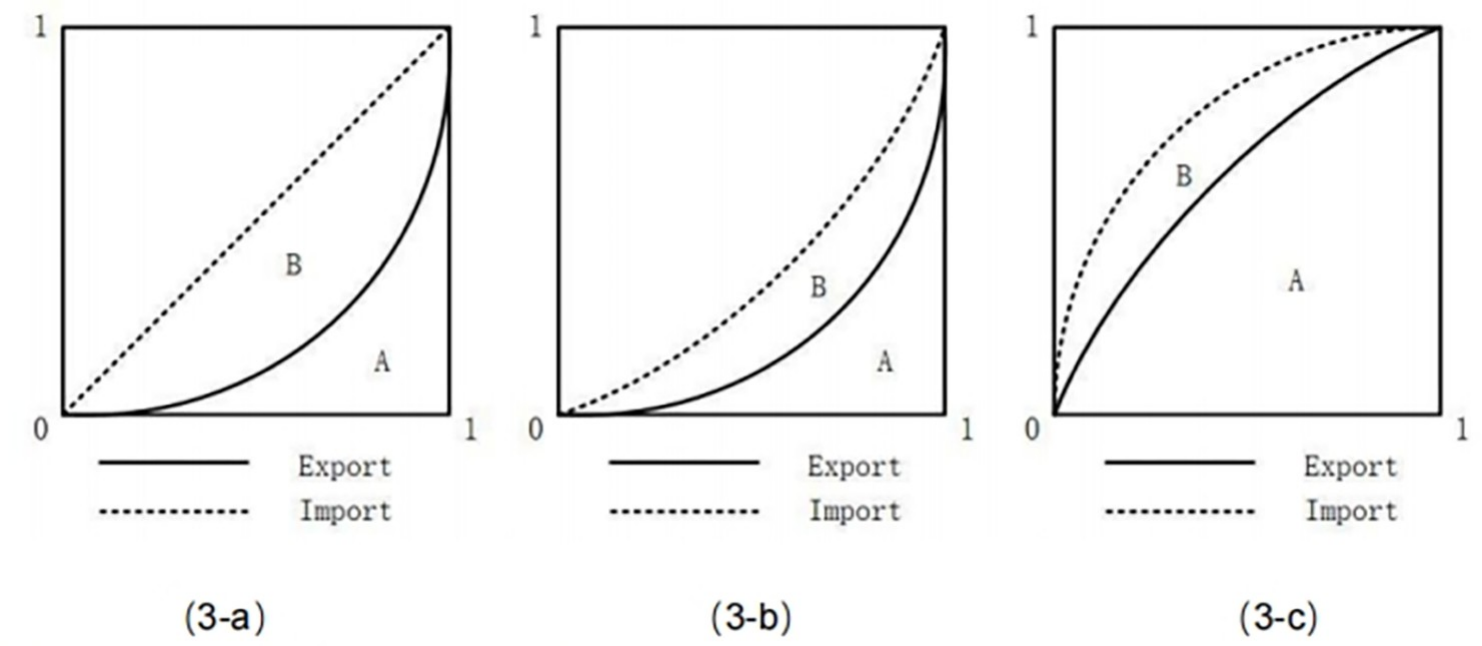
Factor analysis of international surplus value grabbing capacity.

Taking Fig 3 as an illustrative example, we observe that the organic capital composition of the export sector remains unchanged when comparing (3-b). However, in contrast to (3-b), the organic capital composition of the import sector in (3-a) is lower, resulting in a lower level of international division of labour compared to (3-a). This discrepancy arises from the fact that (3-a) captures a greater amount of international surplus value than (3-b), indicated by a larger area B relative to constant area A. Similarly, when comparing with (3-c), although there is no difference in the organic composition index of capital between import and export sectors, the organic capital composition in the export sector of (3-c) is lower than that of (3-b), leading to a reduced level of international division of labour compared to (3-a). The reason for this lies in the fact that while capturing equal amounts of international surplus value, scenario (3-b) incurs smaller costs than scenario (3-c), represented by a smaller area A given an identical area B.

## 4 Using empirical evidence: Reexamining the status of China’s international division of labour

### 4.1 Data sources

The data employed in this study are derived from two databases: the UN Comtrade Database and the National Accounts Main Aggregates Database (NAMA), as provided by the United Nations Statistical Division. The Comtrade database provides data on total export and import trade for all available HS92 six-digit code-classified commodities from 1993 to 2016, presented in terms of current price in US dollars. The time series encompasses a period of 24 years. However, due to factors such as war, self-isolation, poverty, and other reasons, some countries have incomplete or partially missing data, rendering it infeasible to collect complete data for all countries. Nevertheless, as the missing data pertain to relatively small countries, their impact on the overall analysis is minimal. In order to address the fluctuations observed within each year’s value items while examining the status of international division of labour using ordinal methods, it is necessary to mitigate any adverse effects that may arise from such fluctuations being overlooked. Furthermore, the GDP per capita figures for the corresponding years and respective countries were sourced from the NAMA database, based on current dollar prices. In order to reproduce the results of this study, we upload the data used in this paper as supporting information. See Supporting information S1–2 File for details. (dta)

### 4.2 Analysis of empirical results

#### 4.2.1 Longitudinal comparison

**Fig 4:**
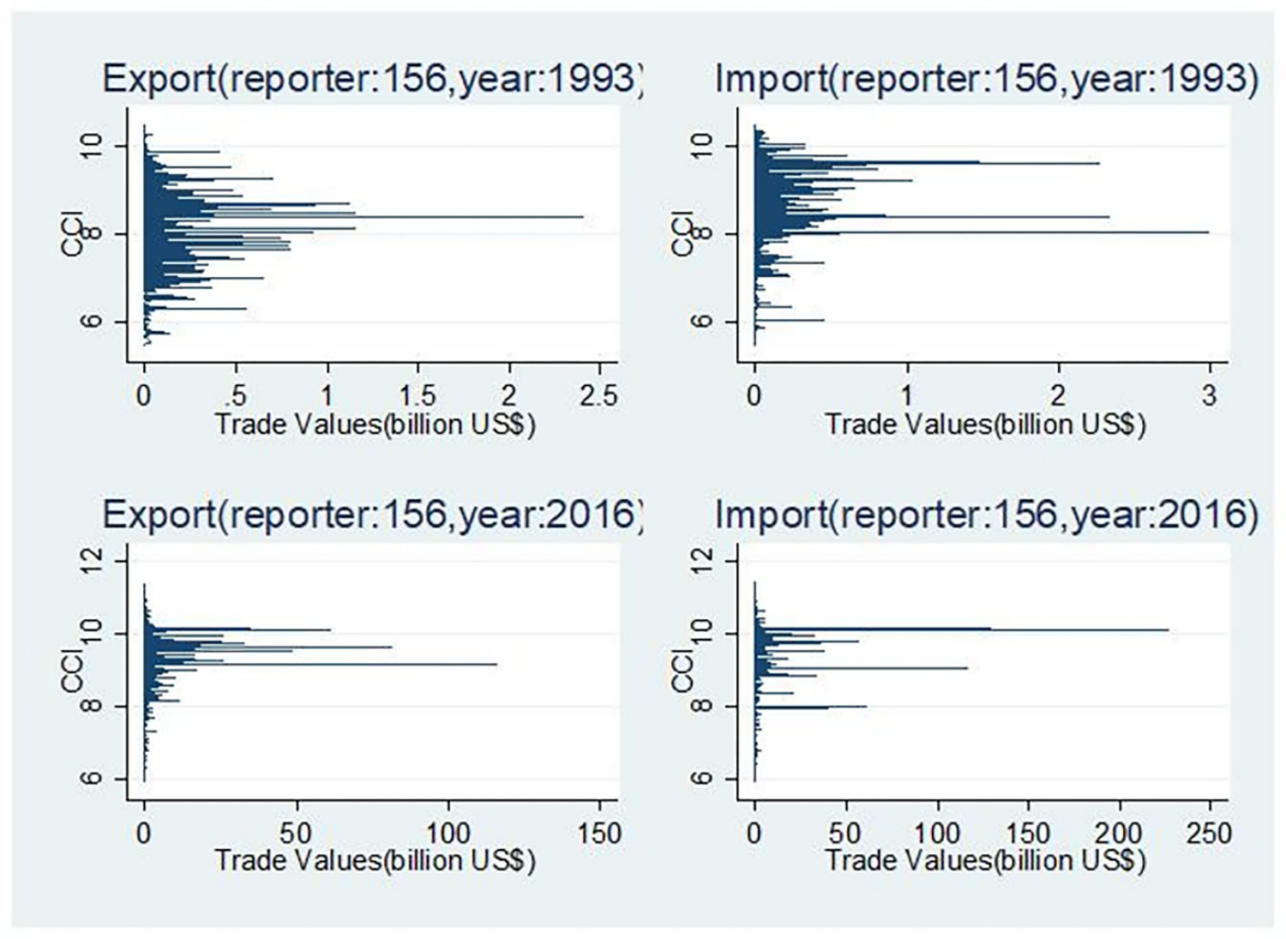
Distribution of capital organic composition in China’s import and export sectors.

Due to spatial limitations, Fig 4 illustrates solely the organic composition distribution of capital in China’s import and export sectors for the years 1993 and 2016. In order to facilitate a comparable analysis between the two sectors on an equal footing, the horizontal and vertical axes have been transformed. The former has been changed to import/export volume, while the latter has been changed to the commodity capital organic composition index. From the perspective of the export sector, there has been an increase in the organic composition of capital from low to high during this period. In 1993, the data exhibited a normal distribution, with the majority of commodities displaying an organic capital composition index below or around 10 (with an average of approximately 8). However, there has been a shift towards higher values since that time, with a considerable number of commodities exhibiting an organic capital composition above 10 and averages well above 8.

With regard to the organic composition of capital in the import sector, there has been a relatively insignificant change over a period of 24 years. In both 1993 and 2016, the data deviate from normal distribution, exhibiting a skew towards higher values. While there is a slight increase in the mean, this is not statistically significant. This finding corroborates Fan’s (2006) conclusion that China’s export technology exhibits an increasing trend in added value from low to high, while China’s import technology consistently demonstrates high added value [10]. Moreover, a comprehensive comparison of the import sector with the general sector reveals that the organic capital composition in imports exceeds that of exports. However, it is noteworthy that this gap has been gradually narrowing. In conclusion, it can be stated that China’s export sector is continuously improving its capital organic composition while maintaining a consistently high level for the import sector. However, there is still some ambiguity regarding the extent to which these two sectors converge.

**Fig 5:**
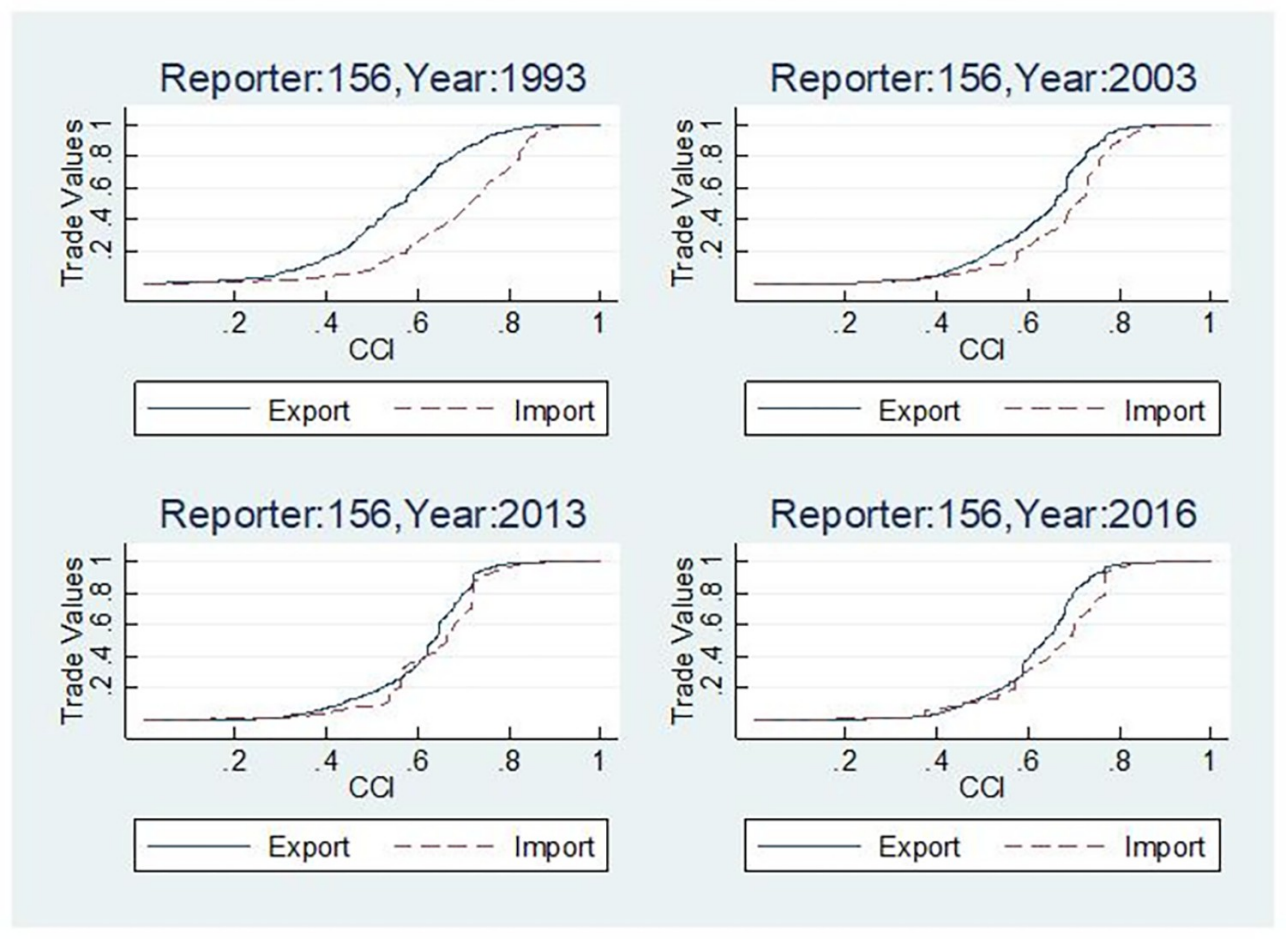
Cumulative distribution curve of organic composition of capital in China’s import and export.

Due to spatial limitations, Fig 5 exclusively presents the cumulative distribution curves of the organic composition of capital in China’s import and export sectors for the years 1993, 2003, 2013, and 2016. It is evident that throughout each year, the capital organic composition of the export sector has consistently remained lower than that of the import sector. This further corroborates the established pattern of exporting low-technology goods while importing high-tech goods. Nevertheless, on a broader scale, this disparity is gradually narrowing over time. However, there still exists a substantial gap between the two cumulative distribution curves depicting the organic composition of capital in both sectors during 1993–2016. Furthermore, while this gap had significantly diminished, it still represents a notable discrepancy. Nevertheless, fluctuations were observed during this process, as evidenced by a slight increase in the gap between 2013 and 2016.

**Fig 6:**
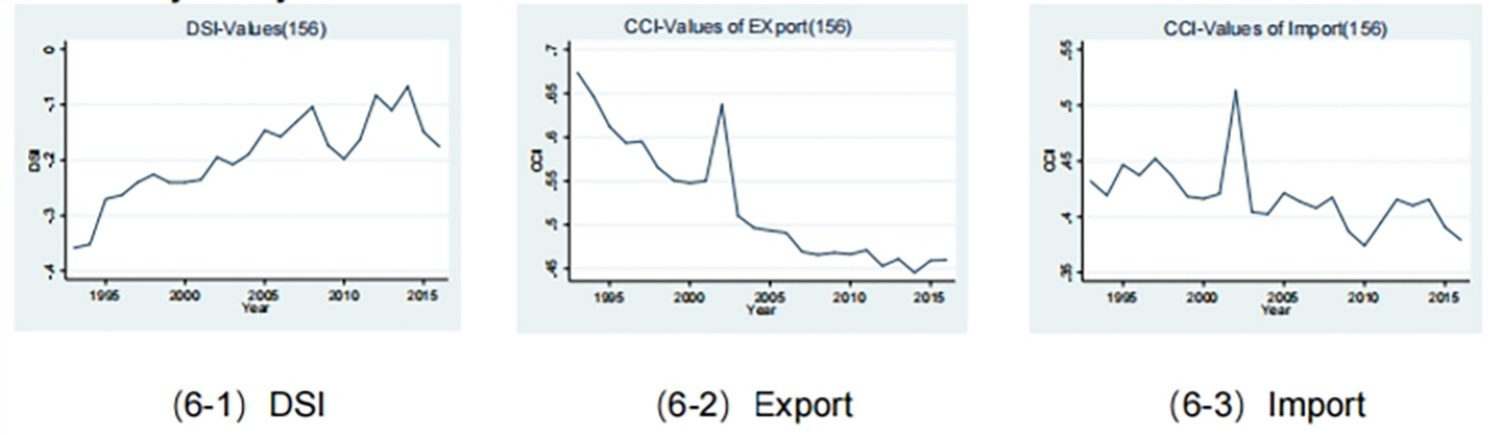
Time series of the division status index and the index of the organic composition of capital in the import and export.

The empirical evidence presented in Fig 6 serves to confirm the increasing trend of China’s export sector’s organic capital composition, as previously observed. As illustrated in Fig 6–2, the organic capital composition index of the export sector displays a downward-sloping curve, whereby a lower index value denotes a higher level of organic capital composition. From 1993 to 2016, with the exception of a notable rebound in 2002 (index value: 0.64), the index consistently declined from 0.67 to reach 0.46 by the end of this period. This decline can be attributed to a number of factors, including potential institutional shocks associated with China’s accession to the World Trade Organization (WTO) during that period. It is noteworthy that the abrupt dismantling of trade barriers upon China’s accession to the WTO led to an increase in low-technology value-added exports, which subsequently resulted in a decline in the overall export sector’s capital composition.

Furthermore, it can be observed from Fig 6–3 that the organic composition of capital in the import sector continues to increase, albeit at a gradual pace. The downward-sloping curve depicted by the index is relatively gentle. The index demonstrated a persistent decline from 0.43 in 1993 to 0.38 in 2016, with a notable resurgence in 2002, reaching 0.51. This resurgence can be attributed to China’s efforts to reduce import costs in advance of its accession to the WTO. Moreover, in addition to high-tech value-added goods, China imported a considerable quantity of medium and low-tech value-added goods during that period, which contributed to a reduction in the overall organic composition of capital within the import sector.

Following a comprehensive examination of the capital organic composition of both the export and import sectors, we have obtained the time series of the division status index, as illustrated in Fig 6–1. The division status index for China demonstrates an upward trajectory, rising from -0.36 in 1993 to -0.18 in 2016. Nevertheless, despite this growth, China’s status as a low participant in the international division of labour remains unchanged. Its index consistently falls below zero, indicating that surplus value continues to be captured by developed countries. The continuous rise in China’s international division of labour status index can be attributed to two factors. Firstly, there is a persistent increase in the organic composition of capital within the export sector. Secondly, this increase occurs at a faster rate than that observed within the import sector, resulting in a narrowing gap between their respective indexes from -0.242 to -0.081. Additionally, an intriguing phenomenon has been identified whereby China’s international division of labour status has experienced oscillations over recent years. In 2009, China experienced a sudden decline in its position within the international division of labour status, with a value of -1.04 compared to the previous year’s value of -1.74. This decline reached its nadir at -1.98 during 2010, before exhibiting a slight rebound in 2011, though without any substantial improvement. By 2012, the index had returned to a positive trajectory, reaching a value of -0.083. However, it then experienced an unexpected decline, falling to -0.150 by 2015.

Additionally, it is evident that the export sector demonstrates a relatively consistent performance, whereas fluctuations are predominantly observed in the import sector. This aligns with the prevailing international division of labour. The index exhibited considerable fluctuations in 2009 and 2015, reaching its lowest point in 2010 and 2016, respectively. It is noteworthy that there was a sudden surge in the organic composition of capital within the import sector during these years, which may have been linked to the subprime crisis and subsequent recovery. As a consequence of the subprime crisis, export prices from developed nations declined. China was therefore able to capitalise on this opportunity by procuring goods with a higher organic capital composition at a comparatively lower cost. Subsequently, China was able to recuperate through industrial structural adjustments. Concurrently, there has been a transition in China’s economic structure, with a shift away from the production chain towards higher-end consumption sectors that are heavily reliant on imports. This adjustment has consequently resulted in an increase in the organic composition of capital within the import sector itself. China is currently situated within a distinctive phase, marked by dual domestic and foreign structural adjustments and a transformation in growth patterns. These factors have the potential to significantly influence the future trajectory of the international division of labour.

### 4.2.2 Horizontal comparison

The vertical comparison of China’s international division of labour status has been discussed above; however, there is still a lack of horizontal comparison. Although the observed trend towards improvement in the international division of labour status index is encouraging, it remains unclear whether this will result in a corresponding shift in the country rankings. In light of the ongoing reform of the international division of labour pattern, it seems plausible that the international division of labour status index for all countries could converge towards zero. This suggests that the index for countries with low international division of labour status will increase, moving closer to zero, while countries with high international division of labour status will see their index decrease towards zero. However, a corresponding subdivision in statistical classification is lacking when transitioning from horizontal specialisation between products to vertical specialisation within products. In its original form, both the import and export sectors included repeated statistics on common goods, which resulted in a gradual narrowing of the gap between these two sectors over time. It thus follows that, although the pattern of international division of labour may remain unchanged, the associated index can fluctuate either upwards or downwards. Consequently, an exclusive reliance on changes observed in the time series data for the international division of labour status index may not provide an accurate assessment or understanding of a country’s actual position within global divisions of labour. It is therefore imperative to conduct a comprehensive horizontal comparison in order to gain insight into China’s evolving position within global divisions.

**Fig 7:**
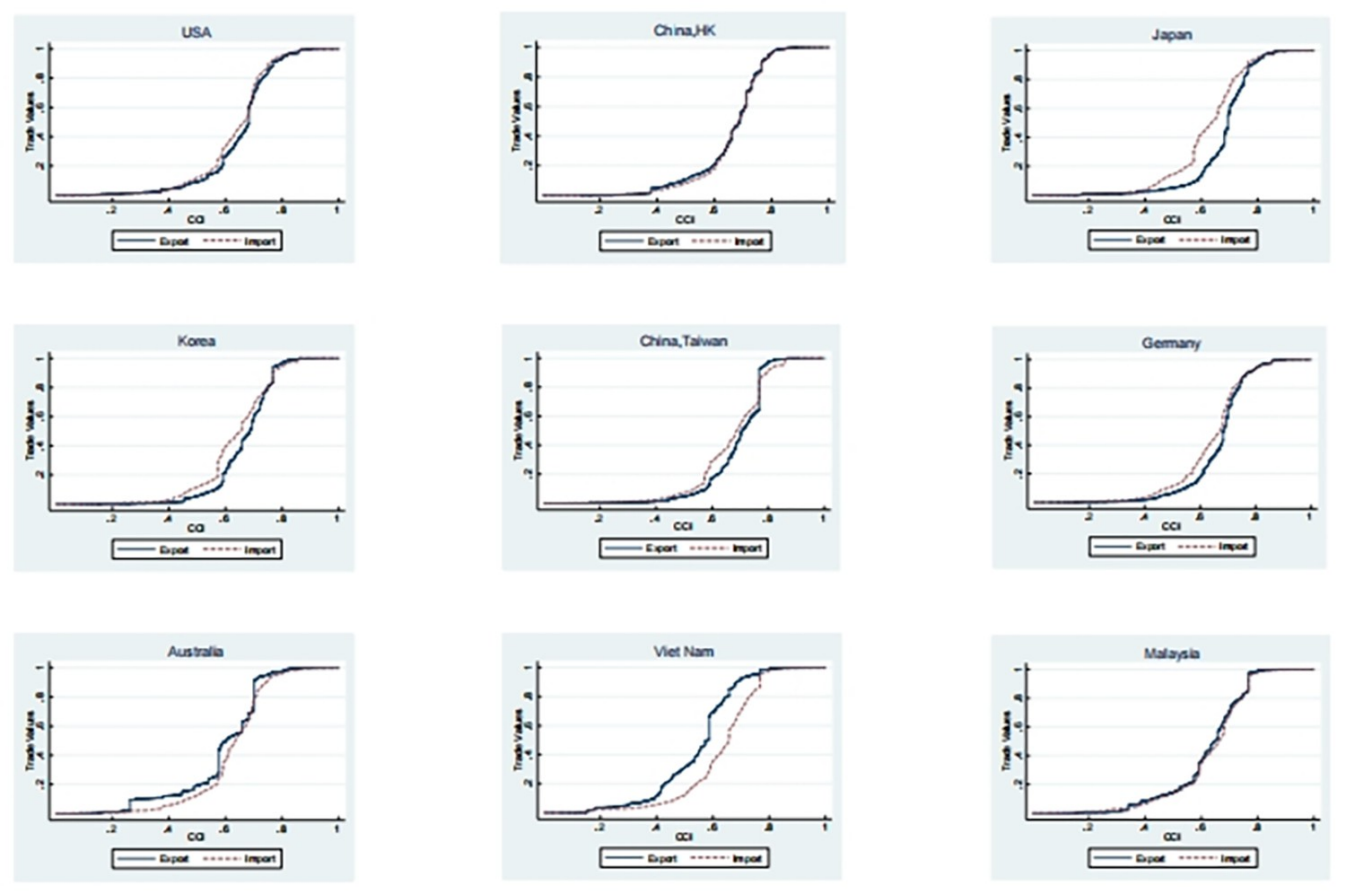
Cumulative distribution of import and export capital organic composition index of some of China’s trading partners in 2016.

It would be beneficial to undertake an analysis of the international division of labour status of China’s principal trading partners. Due to limitations in the available space, only nine countries or regions are included in this analysis. In 2016, the nine countries or regions with the highest total import and export volumes with China were the United States, Hong Kong, Japan, South Korea, Taiwan, Germany, Australia, Vietnam and Malaysia. The list includes both developed and developing countries. Fig 7 illustrates the cumulative distribution curves of the index of the organic composition of capital in the import and export sectors for the aforementioned nine countries or regions. These nine economies can be broadly classified into the following categories: Firstly, there are countries or regions that rely on the capture of surplus value from other countries. This includes Japan, Germany, South Korea and Taiwan. Secondly, the United States is a typical country or region that does not rely on the capture of surplus value from other countries. Thirdly, there are countries or regions that experience the appropriation of surplus value by other nations. To illustrate, as labour-intensive industries relocate to Southeast Asia, Vietnam has also assumed a considerable share of sectors such as clothing, footwear, light electronics, and traditional industries like rice, coffee, and aquatic products. These industries display a markedly low capital organic structure. Similarly, Malaysia is confronted with comparable circumstances; however, it has a robust foundation for growth with favourable momentum. In the ten member countries of the Association of Southeast Asian Nations (ASEAN), Malaysia’s per capita gross domestic product (GDP) and intellectual property protection system are the second highest, after Singapore. Consequently, it is gradually overcoming its unfavourable position in the international division of labour. Australia represents an intriguing case within this category due to its status as an originally developed country that should not occupy a lower position in the global labour division system. Nevertheless, an examination of Australia’s commodity export structure elucidates the underlying reasons for this phenomenon. Abundant natural resources exert a dominant influence on the country’s economy, with commodities such as iron ore, coal, natural gas, beef, wheat, and agricultural products occupying a pivotal position. The organic composition of these commodities is relatively low; however, their abundance allows Australia to become one of the few developed countries reliant on extracting surplus value from other nations.

**Fig 8:**
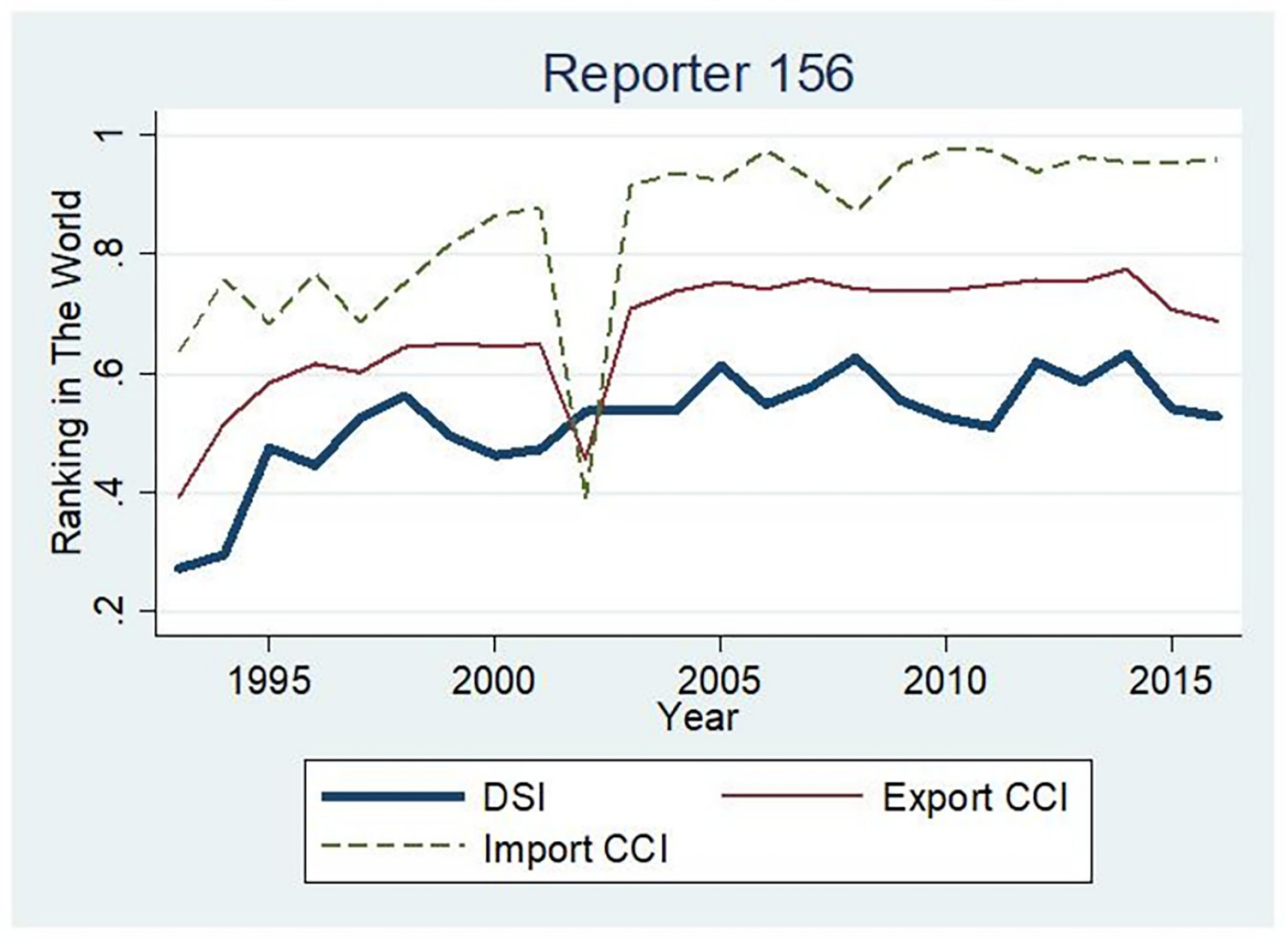
The division status index ranks of China.

Fig 8 illustrates China’s position within the global division of labour. The horizontal axis represents time, while the vertical axis represents the percentage by which China’s high-end degree surpasses that of other countries. A value of 100% indicates that the country in question exceeds all others, whereas a value of 0% implies that it is not superior to any country. Higher values indicate a more robust position within the international division of labour. From the figure, it can be observed that China’s international division of labour status has undergone two distinct stages: an initial period of rapid progress, followed by a subsequent period of weak growth. The percentage increased from 27.3% in 1993 to 56.2% in 1998, but experienced only marginal changes over five years, reaching only 52.8% in 2016. This placed China as the world’s 69th (out of one hundred and forty-four countries and regions surveyed). The composition of organic capital within the import sector demonstrated a notable increase, rising from 63.6% in 1993 to its current level of 95.8%. This places the country in seventh position globally among the nations studied. While the organic capital composition within the export sector also experienced substantial growth, it consistently remained lower than that observed within imports. This increased from 39.3% in 1993 to 68.8% by the end of 2016, and currently ranks 46th globally.

In conclusion, China continues to be exploited by developed countries for surplus value, and its position in the global division of labour remains relatively low. China’s excessive reliance on low-end value chain processing trade has resulted in a lagging capital-organic composition within its export sector relative to its import sector. Presently, China is confronted with mounting challenges in its engagement with the international labour division system. On the one hand, the United States’ reindustrialisation policy aims to reclaim middle and low-end value chains domestically. Conversely, countries such as the European Union, Japan, and South Korea have shifted their industries towards Southeast Asia. Consequently, China is excluded from the current international labour division system. A further deterioration in the capital organic composition within China’s import sector would result from a loss of production capabilities and a concomitant increase in the import of high-grade and high-tech consumer goods. This would have a detrimental impact on China’s status in the global labour division system. In light of the ongoing restructuring of the international system of labour division, it is of paramount importance for China to undertake a strategic realignment of its production structure, redirecting growth momentum in a manner that aligns with the evolving global landscape. This necessitates the establishment or active participation in a novel domestic and international labour division framework.

## 5 Conclusions and recommendations

### 5.1 Conclusions

This paper employs Marx’s theory of international value to develop a methodology for assessing the extent of international division of labour. It utilises a comprehensive data set comprising HS92 six-digit code classified commodities from diverse countries between 1993 and 2016. This enables a vertical and horizontal comparative analysis of China’s level of international division of labour. The findings lead to the following conclusion:

1. The observed pattern of a lower organic composition of capital in China’s export sector relative to the import sector remains unchanged. This indicates that China remains at the lower end of the international division of labour, with surplus value continuing to accrue to developed countries.
2. The organic composition of capital in China’s import sector has consistently exhibited a higher value, while the organic composition of capital in the export sector has consistently demonstrated a lower value than that observed in the import sector. This demonstrates that China has consistently relied on the global division of labour system dominated by developed countries. Additionally, it illustrates that China’s high-tech consumer goods are also heavily reliant on imports from abroad.
3. There is a discernible upward trajectory in the organic composition of capital in China’s export and import sectors, with the former exhibiting a markedly accelerated rate of improvement in comparison to the latter. This indicates that the absolute value of China’s captured surplus is undergoing a continuous reduction, thereby creating an opportunity to improve the unfavourable position of the international division of labour.
4. An analysis of China’s global ranking in terms of its international division of labour status index indicates that there has been no discernible improvement in this area. While the absolute value of China’s captured surplus has been declining, the enhancement of the country’s status in the global division of labour has been relatively insignificant. This indicates that the reduction in the absolute value of the captured surplus is a global phenomenon, rather than a phenomenon specific to China. It is noteworthy that as the organic composition of capital increases, the proportion of variable capital in the total social capital declines, leading to a reduction in surplus value and, consequently, a decline in the profit rate [30,31]. This was also demonstrated in the findings of Basu et al. (2019) and Rotta et al. (2024).
5. The overall growth of China’s international division of labour status index is relatively weak. The period in question can be roughly divided into two stages. Between 1993 and 1998, there was a certain increase, but after nearly 20 years, the international division of labour status index ranking basically did not increase. Notwithstanding China’s accession to the WTO, the country has not witnessed any discernible positive effects with respect to its international division of labour status.

### 5.2 Recommendations

The unequal exchange in the international division of labour is a consequence of the globalisation of capitalist production relations, rather than the result of the specific actions of individual countries or groups. Even China, if its capital organic composition meets certain conditions, will appropriate the international surplus value of other countries in the international market. In the context of capitalist globalisation, it is inevitable that countries will be subject to the control of the law of capitalist value, resulting in the realisation of unequal exchange through the transfer of international surplus value. It is inevitable that some developing countries will remain at the lower end of the international division of labour, and thus vulnerable to exploitation by more economically advanced nations.

The most fundamental measure to improve the status of the international division of labour of the vast number of developing countries, such as China, is to align national and international values. One potential solution is the establishment of a global body with international legal authority, operating beyond the boundaries of the nation-state, with the objective of raising wages in less economically developed countries. However, this is a highly implausible proposition. A further avenue for exploration, proposed by other scholars, is to return to specific countries and minimise the transfer of domestic value to the international market. The prevailing view is that reliance on foreign markets and international resources, coupled with greater integration into the international division of labour system, represents a viable strategy. To illustrate, a new phase of international industrial transfer should be initiated. It is anticipated that the forthcoming phase of global international industrial transfer will be dominated by the service industry. Consequently, China has the potential to enhance its status within the international division of labour by undertaking offshore service outsourcing [32]. An additional example is the reliance on multinational companies to reinforce infrastructure and enhance service quality, with the objective of attracting multinational companies to establish high-value added upstream operations in China [33]. An additional example is the promotion of the upgrading of processing trade and the vigorous development of export-oriented supporting industries [34].

It is recommended that these views be considered and implemented, but this paper puts forth an alternative proposal: to enhance the status of the international division of labour through the development of domestic regional cooperation. The international division of labour system is characterised by a concentration of power in developed countries, which gives rise to a number of inherent deficiencies. The reason for this is that the mechanisms that promote technological progress, such as specialisation, competition and knowledge spillover, are either ineffective or at least suppressed in this system. Consequently, it is challenging for developing countries to undergo evolutionary and upgrading processes through this system. In other words, developing countries are susceptible to becoming enmeshed in an inferior international division of labour. This illustrates why China’s accession to the WTO has not led to a notable enhancement in the status of international division of labour. Furthermore, the growth rate of the division status index in the global ranking has not accelerated as much as it did prior to the country’s accession to the WTO.

In light of the increasingly passive role of the international division of labour, it may be beneficial to consider the establishment of a domestic division of labour system. As a consequence of the initial accumulation within the international division of labour system, China is now in a position to establish a domestic division of labour system. The initiative to establish a domestic division of labour system, with the objective of fostering interregional interconnectivity between the eastern coastal areas and the central and western regions, is driven by the intention to activate three core mechanisms: specialisation, competition and knowledge spillover. This is with a view to enabling the domestic division of labour system to evolve and undergo progressive upgrading, thereby enabling it to transition from a primary to an advanced division of labour system. The activation of the three mechanisms is contingent upon the intrinsic merits of the domestic division of labour system, which permits a relatively free circulation of production factors in comparison to the international system. This provides the material basis for specialisation, competition and knowledge spillover. The construction of a domestic division of labour system should be accompanied by a systematic transfer of labour-intensive industries to the central and western regions [35], with the coastal areas beginning to cultivate technology-intensive industries. In this manner, the eastern, central, and western regions have established a fundamental domestic division of labour system between horizontal products. As the division of labour system continues to evolve, it can eventually evolve into vertical products. Furthermore, as technology progresses, the organic composition of capital in the export sector will inevitably be enhanced [36], thereby further improving the status of the international division of labour.

## Supporting information

S1 File (ZIP)
